# Supplementary material for: Initial Experience of Metabolic Imaging with Hyperpolarized [1-13C]pyruvate MRI in Kidney Transplant Patients
Source: ArXiv. 2024 Sep 10:arXiv:2409.06664v1. Preprint. [Version 1] (PMC11419194)
Supplement: Supplement 1 [file NIHPP2409.06664v1-supplement-1.pdf]

Supplementary Information

Tables

Table S1: Apparent diffusion coefficient (ADC) and R_2* of kidney allografts and native non-tumor bearing kidneys from patients with renal cell carcinomas.

**Participants with kidney Allograft**		
Participant	ADC ($\times10^{-3}$mm^2/s)	R_2* (s^{-1})
#1	N/A	21.89±14.59
#2	2.10±0.11	14.15±9.21
#3	1.78±0.26	N/A
#4	N/A	14.41±8.62
#5	1.76±0.08	24.70±14.71
#6	1.92±0.09	17.61±10.16
**RCC patients with native non-tumor bearing kidneys**		
#1	1.99±0.09	N/A
#2	1.94±0.10	15.03±8.06
#3	2.07±0.10	27.24±12.77
#4	2.00±0.09	22.96±9.81
#5	1.92±0.09	14.44±9.63

Note: N/A: Not available, either due to not acquired (ADC) or insufficient signal-to-noise ratio (SNR) for quantification (R_2*).

Table S2: Comparison of lactate-to-pyruvate ratio and bicarbonate-to-pyruvate ratio of kidney allografts and native non-tumor bearing kidneys with "mean time" correction and without "mean time" correction.

**Participants with kidney Allograft**				
Participant	**With Mean Time Correction**		**Without Mean Time Correction**	
	Lactate-to-Pyruvate Ratio	Bicarbonate-to-Pyruvate Ratio	Lactate-to-Pyruvate Ratio	Bicarbonate-to-Pyruvate Ratio
#1	0.42±0.04	0.05±0.01	0.42±0.04	0.05±0.01
#2	0.38±0.03	0.040±0.03	0.21±0.01	0.03±0.00
#3	0.85±0.07	0.11±0.01	0.85±0.07	0.11±0.01
#4	0.54±0.05	N/A	0.24±0.02	N/A
#5	0.56±0.04	0.06±0.01	0.38±0.04	0.04±0.04
#6	0.32±0.03	0.04±0.00	0.32±0.03	0.04±0.00
**RCC patients with native non-tumor bearing kidneys**				
#1	0.39±0.03	N/A	0.28±0.02	N/A
#2	0.25±0.04	N/A	0.19±0.01	N/A
#3	0.25±0.02	N/A	0.22±0.02	N/A
#4	0.37±0.03	N/A	0.37±0.03	N/A
#5	0.39±0.02	0.06±0.01	0.39±0.03	0.06±0.01

Note: N/A: Not available.

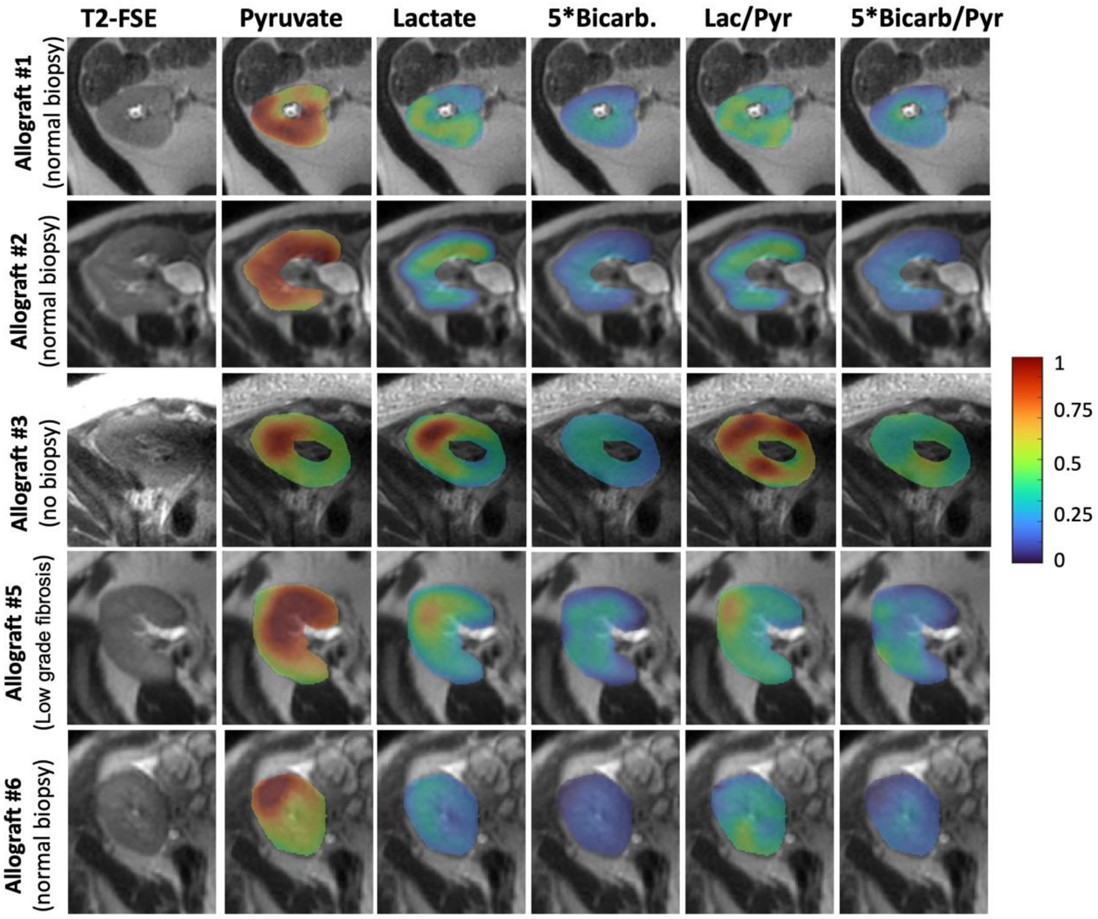

Figure S1 ^{13}C pyruvate, lactate, bicarbonate and metabolite ratio images from five kidney transplant participants. All area under the curve (AUC) images were normalized by the kidney pyruvate AUC. Due to the low signal level of bicarbonate, all bicarbonate images are shown with 5-time signal amplified.

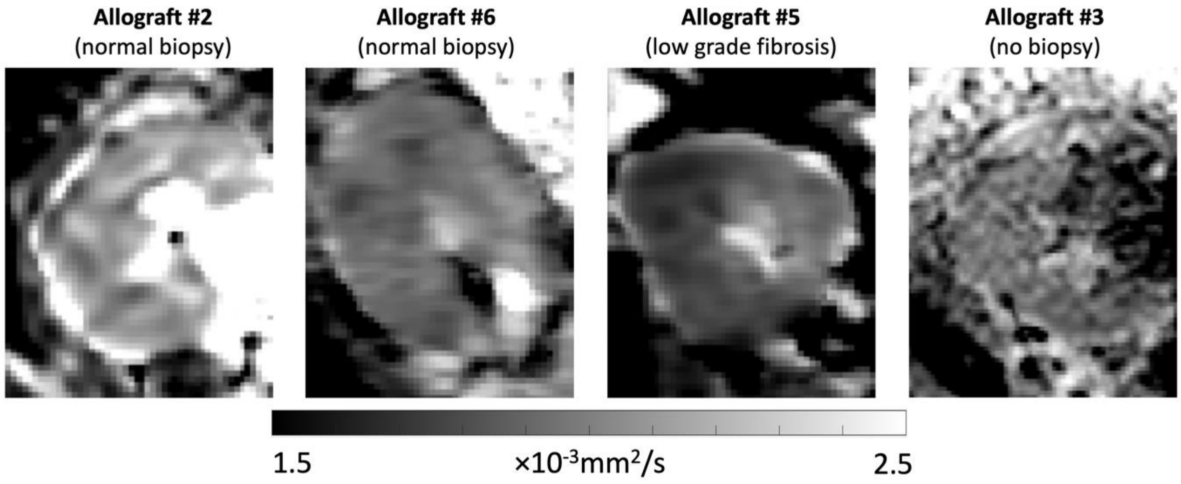

Figure S2. Apparent diffusion coefficient (ADC) maps from four kidney allografts.

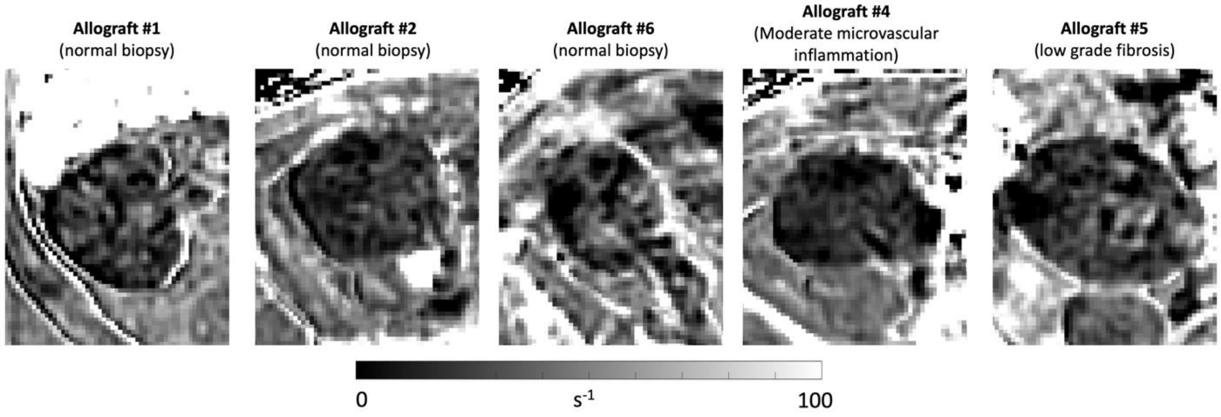

Figure S3. R_2* maps from five kidney allografts.
